# mTOR signaling and its roles in normal and abnormal brain development

**DOI:** 10.3389/fnmol.2014.00028

**Published:** 2014-04-23

**Authors:** Nobuyuki Takei, Hiroyuki Nawa

**Affiliations:** Department of Molecular Neurobiology, Brain Research Institute, Niigata UniversityNiigata, Japan

**Keywords:** mTORC1 signaling, translational control, protein synthesis, BDNF, CNS neurons, amino acids, TSC/mTOR, brain malformation

## Abstract

Target of rapamycin (TOR) was first identified in yeast as a target molecule of rapamycin, an anti-fugal and immunosuppressant macrolide compound. In mammals, its orthologue is called mammalian TOR (mTOR). mTOR is a serine/threonine kinase that converges different extracellular stimuli, such as nutrients and growth factors, and diverges into several biochemical reactions, including translation, autophagy, transcription, and lipid synthesis among others. These biochemical reactions govern cell growth and cause cells to attain an anabolic state. Thus, the disruption of mTOR signaling is implicated in a wide array of diseases such as cancer, diabetes, and obesity. In the central nervous system, the mTOR signaling cascade is activated by nutrients, neurotrophic factors, and neurotransmitters that enhances protein (and possibly lipid) synthesis and suppresses autophagy. These processes contribute to normal neuronal growth by promoting their differentiation, neurite elongation and branching, and synaptic formation during development. Therefore, disruption of mTOR signaling may cause neuronal degeneration and abnormal neural development. While reduced mTOR signaling is associated with neurodegeneration, excess activation of mTOR signaling causes abnormal development of neurons and glia, leading to brain malformation. In this review, we first introduce the current state of molecular knowledge of mTOR complexes and signaling in general. We then describe mTOR activation in neurons, which leads to translational enhancement, and finally discuss the link between mTOR and normal/abnormal neuronal growth during development.

## INTRODUCTION: IDENTIFICATION OF TOR AND mTOR

Mammalian target of rapamycin (mTOR) controls growth and metabolism by activating anabolic processes and suppressing catabolic processes. Dysregulation of mTOR signaling induces various disorders including cancer, diabetes, obesity, cardiovascular disease, inflammation, and neurodevelopmental and neurodegenerative disorders. Several inhibitors of mTOR have been developed and are now clinically approved. Recently, remarkable progress has been made in understanding the molecular nature of mTOR signaling and its biological significance. Here we overview the signaling network of mTOR cascades with special emphasis on translational control. We also focus on the normal and abnormal growth of neurons regulated by mTOR complex 1 (mTORC1).

Mammalian target of rapamycin is a 289-kDa serine/threonine protein kinase that is, as indicated by its name, a target molecule of the immunosuppressant rapamycin. Rapamycin is an anti-fungal macrolide compound isolated from soil bacterium, *Streptomyces hygroscopicus*, in Rapa Nui (Easter Island; Vézina et al., 1975). The 12-kDa FK506-binding protein (FKBP12) was first identified as a rapamycin-binding protein (Heitman et al., 1991a; Schreiber, 1991). However, yeast genetic screening revealed that FKBP12 was not a functional TOR because disruption of *FRP1* (FKBP12 coding gene in the budding yeast *Saccharomyces cerevisiae*) did not cause growth inhibition/toxicity. This suggested that FKBP12 was likely a co-factor in executing the rapamycin action. Screening of rapamycin-resistant yeast mutants led to the identification of genes *TOR1* and *TOR2* as molecular targets of rapamycin-FKBP12 complex and functional targets of rapamycin (Heitman et al., 1991b). Subsequently, four groups identified rapamycin targets in mammalian cells: mTOR, FRAP (FKBP12 and rapamycin associated protein), RAFT1 (rapamycin and FKBP12 target 1), and RAPT1. Although recent database nomenclature defined the abbreviation MTOR as the “mechanistic TOR” in mammals, many researchers still use the name mTOR (Hall, 2013).

## mTOR COMPLEX: COMPONENTS OF mTORC1 AND mTORC2

**FIGURE 1:**
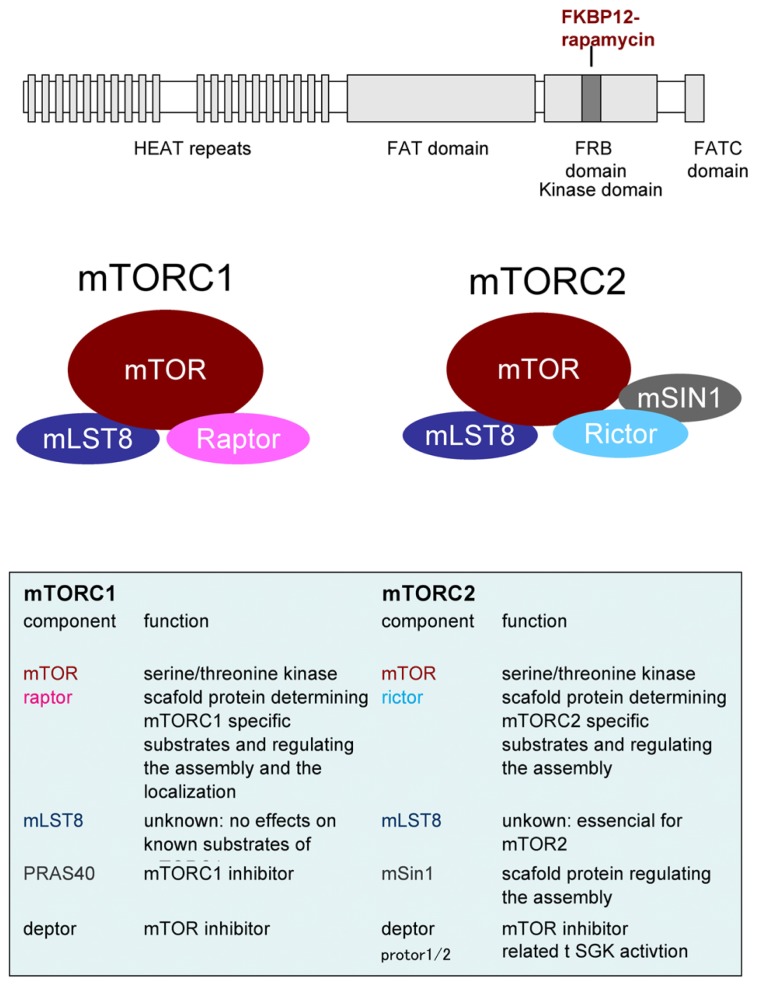
**Upper panel.** Domain structure of mTOR. HEAT: **H**untington **E**longation Factor 3 PR65/**A T**OR, FAT: **F**RAP **A**TM **T**TRAP, FRB: **F**KBP12-**R**apamycin **B**inding. Middle and lower panel: Components of mTOR complexs.

Whereas yeast expresses two TOR molecules, TOR1 and TOR2, only one TOR homolog, mTOR, exists in mammals. mTOR nucleates two distinct complexes called mTORC 1 and 2 with several interacting proteins; these complexes have different functions. For example, mTORC1 governs cell growth, metabolism and cell cycle, whereas mTORC2 controls cell survival and cytoskeleton organization (see review by Laplante and Sabatini, 2012). The composition of mTORC1 and mTORC2 is shown in **Figure 1** and detailed below. The main characteristic component of mTORC1 is raptor (Hara et al., 2002; Kim et al., 2002). Raptor is a scaffold protein that regulates complex assembly in addition to substrate recognition, that is to say, it determines downstream signaling of mTORC1. Likewise, rictor is a crucial binding partner of mTOR to make it function as mTORC2 (Jacinto et al., 2004; Sarbassov et al., 2004). In fact, as discussed below, mTORC1 and mTORC2 functions are impaired by the knockout/knockdown of raptor or rictor, respectively. The mammalian lethal with sec13 protein 8 (mLST8) is thought to be a requisite for mTORCs activities and mSin1 is thought to be a scaffold for mTORC2. In addition, deptor and PRAS40 are inhibitor molecules for mTORCs. Several other molecules are reported to be involved in these complexes but their roles are still unclear. Rapamycin, in binding with FKBP-12, interacts with mTOR on its FKBP12-rapamycin binding (FRB) domain and inhibits mTORC1 activity. Many, but not all (Kang et al., 2013), of the substrates and biological processes controlled under mTORC1 are rapamycin-sensitive. mTORC2 activity, in contrast, has been thought to be rapamycin-insensitive. Recently structural analysis of mTOR gives us an important insight into the mechanism of rapamycin action (Yang et al., 2013). The work predicts that FKBP12-rapamycin complex bound FRB domain may come close to mLST8 thus reducing the access of the substrates to active site of mTOR. These structural properties of mTOR may contribute to the preferences of substrates and/or phosphorylation sites by rapamycin inhibition (Choo and Blenis, 2009; Kang et al., 2013). In case of mTORC2, its components may already be bound proximately to FRB domain thus inhibits FRB. This notion is supported by the two findings. Prolonged treatment with rapamycin affects mTORC2 activity (Sarbassov et al., 2006). Rapamycin does not liberate a pre-existing rictor-mTORC but inhibits the interaction of newly synthesized rictor with mTOR. Another finding is that micromolar order of rapamycin inhibits mTORC2 activity independently of FKBP12 (Shor et al., 2008). High dose of rapamycin may allows free-rapamycin that can bind FRB domain and thus inhibits mTORC2. These findings advance our knowledge about the mechanism of the inhibitory action of rapamycin.

## SIGNAL TRANSDUCTION PATHWAYS OF mTORC1

In contrast to the dearth of knowledge about the signaling pathway of mTORC2, the upstream and downstream pathways of mTORC1 are much better understood. mTORC1 integrates several intracellular and extracellular cues, and transduces divergent downstream events. Growth factors and amino acids are the best-characterized extracellular stimuli that activate mTORC1. In addition, cellular energy status, oxygen/hypoxia and stressors regulate mTORC1 activity. In the central nervous system (CNS), neurotransmitters, neuromodulators, and hormones are reported to activate mTORC1.

### UPSTREAM SIGNALING AND STIMULANTS OF mTORC1

Growth factors bind to and activate receptor tyrosine kinases (RTKs). The RTK to Akt (aka PKB) pathway has been well investigated so far. Then, tuberous sclerosis complex 1 (TSC1; aka hamartin) and TSC2 (aka tuberin) have been identified as links between Akt and mTOR (Gao et al., 2002; Inoki et al., 2002; Tee et al., 2002). Akt phosphorylates TSC2 and causes it to dissociate from TSC1. Dissociated TSC2 is captured by 14-3-3 and is prevented to form the complex (Li et al., 2002; Nellist et al., 2002; Shumway et al., 2003). Phosphorylated and liberated TSC2 is degraded by ubiquitination (Chong-Kopera et al., 2006; Hu et al., 2008), although the role of 14-3-3 in this process is unclear. The TSC1/2 plays a key role in the activation of mTOR. TSC1/2, together with a third component TBC1D7 (Nakashima et al., 2007) functions as a GTPase-activating protein (GAP) for small G-protein Ras homology enriched in brain (Rheb; Inoki et al., 2003a; Dibble et al., 2012). GTP-bound Rheb, the active form, directly binds to mTORC1 and activates its kinase activity (Long et al., 2005). The GAP accelerates GTP hydrolysis, which inactivates Rheb into its GDP-bound form. In steady state, without growth factor stimulation, unphosphorylated TSC2 maintains a heteromeric interaction with TSC1, and this complex is a suppressor of mTRC1. Until now, the guanine nucleotide exchange factor (GEF) that promotes GDP to GTP conversion of Rheb had not been identified. In addition to Akt, mitogen-activated protein kinase (MAPK), and Rsk inhibit TSC2 thus activating mTORC1. As discussed below, the mutations that causes loss of function of TSCs result in mTORC1 over activation and cause brain diseases.

Tuberous sclerosis complex 2 directly receives many upstream signals by its phosphorylation. The phosphorylation of TSC2 induces both activation and inactivation of mTORC1 depending on the phospho-acceptor amino acid residues. For example, phosphorylation at Thr1462 of TSC2 by Akt (Inoki et al., 2002; Manning et al., 2002) and Ser664 by MAPK (aka Erk; Ma et al., 2007) inhibits TSC1/2 activity thus activating mTORC1, whereas Ser1345 phosphorylation by AMP-activated protein kinase (AMPK) enhances TSC1/2 activity (Inoki et al., 2003b). GSK3β (glycogen synthase kinase) also phosphorylates TSC2 at Ser 1337 and Ser1341 after priming phosphorylation at Ser1345 by AMPK and activates TSC1/2, thus inhibiting mTORC1 (Choo et al., 2006; Inoki et al., 2006).

### STIMULANTS OF mTORC IN NEURONS

#### Growth factors and neurotransmitters

Insulin is the most-characterized growth factor that activates mTORCs through the PI3K/Akt/TSC/Rheb pathway (Avruch et al., 2006). Indeed, insulin and insulin-like growth factor 1 (IGF1) enhance mRNA translation in neurons possibly through mTORC1 (Quevedo et al., 2002; Lee et al., 2005; Chenal et al., 2008). Among several growth factors tested, brain-derived neurotrophic factor (BDNF), the most prominent neurotrophic factor in the CNS (Lewin and Barde, 1996; Nawa and Takei, 2001), has been shown to activate mTORC1 signaling and enhance novel protein synthesis in cortical neurons (Takei et al., 2001, 2004). Interestingly, the effects of BDNF on translation were stronger than that of insulin likely because BDNF activates both Akt and MAPK, whereas insulin activates only Akt in neurons. This makes sense because TSC2 is phosphorylated by both Akt and MAPK. In addition to BDNF and insulin, several growth factors/RTKs (Kim et al., 2008a; Yao et al., 2013) and guidance molecules such as Eph, Slit, and Reelin (detailed in the axonal growth section) have been reported to activate mTORC1 signaling in neuronal cells.

A list of activators of mTORC1 in neurons is shown in **Table 1**. For example, G-protein coupled receptors (GPCRs) such as μ-opioid (Polakiewicz et al., 1998), metabotropic glutamate (Hou and Klann, 2004; Page et al., 2006), and cannabinoid (Puighermanal et al., 2009) receptors activate mTORC1 in neurons. Because GPCRs are known to transduce signals to Akt and/or MAPK, mTORC1 activation triggered by these ligands seems to inhibit TSC2. Other neurotransmitters were reported to possibly activate mTORC1 (meaning that the outputs are sensitive to rapamycin), such as serotonin (5-HT; Casadio et al., 1999; Carroll et al., 2004, 2006), may utilize this pathway.

**Table 1:** Stimulants of mTOR in CNS neurons.

Stimulants	Validation	Responses	Reference
**Growth factors**
BDNF	mTOR kinase activity	Protein synthesis	Takei et al. (2001)
	Substrates phosphorylation		Liao et al. (2007), Manadas et al. (2009)
	Rapamycin	Local protein synthesis	Takei et al. (2004)
	siRNA	Translation elongation	Inamura et al. (2005)
CNTF	mTOR kinase activity, rapamycin	
		STAT3 phosphorylation and activation	Yokogami et al. (2000)
IGF	Rapamycin		
	Substrate phosphorylation	Protein synthesis	Quevedo et al. (2002)
		MCT2 level	Chenal et al. (2008)
Insulin	Rapamycin		
	Substrate phosphorylation	PSD95 level	Lee et al. (2005)
		MCT2 level	Chenal et al. (2008)
Neuregulin	Rapamycin		
		Kv4.2 level, outward K^+^ current	Yao et al. (2013)
VEGF	Substrate phosphorylation		
			Kim et al. (2008a)
**Guidance molecules**
Reelin	Substrate phosphorylation		
	Rapamycin	Dendritic growth	Jossin and Goffinet (2007)
Semapholin-3	Rapamycin		
Netrin-1	Substrate phosphorylation	Growth cone collapse and turning	Campbell and Holt (2001)
Slit-2			
			Piper et al. (2006)
EphrinA1	Substrate phosphorylation		
(Inhibitory action)	Rapamycin	Axon guidance	Nie et al. (2010)
		Protein synthesis	
**Neurotransmitters**
Glutamate	Substrate phosphorylation		
	Rapamycin		Lenz and Avruch (2005)
		Local protein synthesis	Gong et al. (2006)
mGluR agonist	mTOR phosphorylation		
(DHPG)	Rapamycin	LTD	Hou and Klann (2004)
	Substrate phosphorylation		
			Page et al. (2006)
mOpioid R agonist	Substrate phosphorylation		
(DAMGO)	S6K kinase activity		Polakiewicz et al. (1998)
	Rapamycin		
5-HT	Rapamycin		
	eEF2 dephosphorylation	LTF	Casadio et al. (1999)
	Substrate phosphorylation		Carroll et al. (2004)
		Translation	Carroll et al. (2006)
Cannabinoid	Substrate phosphorylation		
(THC)	Rapamycin	Cognitive test	Puighermanal et al. (2009)

Neural activity-dependent regulation is another characteristic feature of mTORC1 activation in the CNS. For instance, the paradigms of synaptic plasticity such as long-term potentiation (LTP) and long-term depression (LTD) often induce mTORC1 activation. mTORC1 activation associates with spatial learning and fear conditioning as well. The direct activator molecules driving these plasticity-related or behavioral paradigms are likely the growth factors or neurotransmitters mentioned above. Indeed, the involvement of BDNF in LTP and metabotropic glutamate receptors (mGluRs) in LTD is well known. Interestingly, Rheb, an upstream activator of mTORC1, was originally found as a growth factor- and an activity-dependent transcript in the brain so that it named “enriched in brain” (Yamagata et al., 1994). This mechanism may also participate in mTORC1 activation by neural activity. The increase of blood flow during learning, as revealed by blood oxygenation level-dependent functional magnetic resonance imaging (BOLD fMRI) may supply enough nutrients such as glucose and amino acids. As mentioned below, these nutrients are other stimulants of mTORC1. More detailed discussions on mTORC1 in synaptic plasticity can be found in other reviews in this issue.

#### Nutrients

Yeast TOR activity is controlled by nutrients. Similarly, amino acids, especially leucine, are other essential extracellular stimuli that activate mTORC1 (Hara et al., 1998) in mammalian cells. Amino acids are not only the building blocks of proteins or intermediates of metabolism but are also evolutionarily conserved inter-cellular signaling molecules in both eukaryotes and prokaryotes. For example, glutamate and GABA are important neurotransmitters in the CNS. Recent reports have answered the long-lasting question, why and how leucine activates mTORC1. The analysis of raptor binding partner (Sancak et al., 2008) and siRNA screening of small GTPases (Kim et al., 2008b) revealed the link between Rag family small G-proteins (RagA–D) and mTORC1. RagA or RagB (~98% sequence similarity) forms a heterodimer with RagC or RagD (~87% sequence similarity). The active conformation is a heterodimer of RagA/B·GTP and RagC/D·GDP. When amino acid availability is sufficient, this complex directly binds to raptor and recruits mTORC1 to the lysosome with “Ragulator (consisting with three proteins, MP1/P14/P18),” which is a GEF for RagA/B. This makes mTORC1 close to and bind to Rheb, an activator of mTORC1 on the lysosomal membrane. A surprising molecule now comes into play – leucyl-tRNA synthetase (LeuRS) – both in mammalian cells (Han et al., 2012) and in yeast (Bonfils et al., 2012). LeuRS is an aminoacyl-tRNA synthetase that loads leucine to tRNA with high fidelity. LeuRS binds to RagD and acts as a GAP. Thus, RagD stays in a GDP-bound form, which is necessary for mTORC1 activation. This may be the mechanism that cells sense leucine and activates mTORC1. However, there are still unsolved questions (Segev and Hay, 2012), such as why among the 20 aminoacyl-tRNA synthetases only LeuRS works in the mTORC1 system. Because other amino acids also activate mTORC1, additional players must be involved.

mTORC1 converges nutrients and growth factor signals, and there is cross-talk between them. Reports suggest that amino acid(s) sufficiency is essential for the insulin-induced activation of mTORC1 in several cell lines (Hara et al., 1998; Campbell et al., 1999). Leucine activates mTORC1 in neurons after influx through system L amino acid transporter (LAT), consisting of LAT1 or LAT2 and 4F2hc (CD98; Ishizuka et al., 2008). Uptake of arginine by cationic amino acid transporters CAT1 and CAT3 has also been reported to activate mTORC1 in neurons (Huang et al., 2007). However, contrary to the above-mentioned essential role of amino acids, starvation of amino acids is less effective on BDNF-induced mTORC1 activation in neurons (Ishizuka et al., 2013). This discrepancy is unclear; however, cell-type specific amino acid contents or autophagy mechanisms may influence the system responses.

mTORC1 signaling in neurons seems to be more sensitive to energy status. For instance, glucose starvation increases the ratio of AMP/ATP. Increased AMP activates AMPK (Witters et al., 2006). AMPK directly phosphorylates TSC2 and enhances TSC1/2 activity, leading to the inhibition of mTORC1 (Inoki et al., 2003b, 2006). Another target to inhibit mTORC1 by AMPK is raptor. Raptor is directly phosphorylated by AMPK at Ser722/Ser792 and this leads to the inhibition of mTORC1 activity (Gwinn et al., 2008). In neurons, AMPK activation completely abrogated growth factor-induced mTORC1 activation and protein synthesis, with TSC2 and raptor phosphorylation (Ishizuka et al., 2013). AMPK is phosphorylated and activated by LKB1 (Woods et al., 2003) and calcium/calmodulin-dependent protein kinase kinase (CaMKK; Hurley et al., 2005). Because CaMKK activity is dependent on calcium, both ionotropic and metabotropic receptor ligands (neurotransmitters) may participate in mTORC1 signaling through the Ca2+-CaMKK-AMPK pathway in neurons.

### DOWNSTREAM SIGNALING OF mTORCs

**FIGURE 2:**
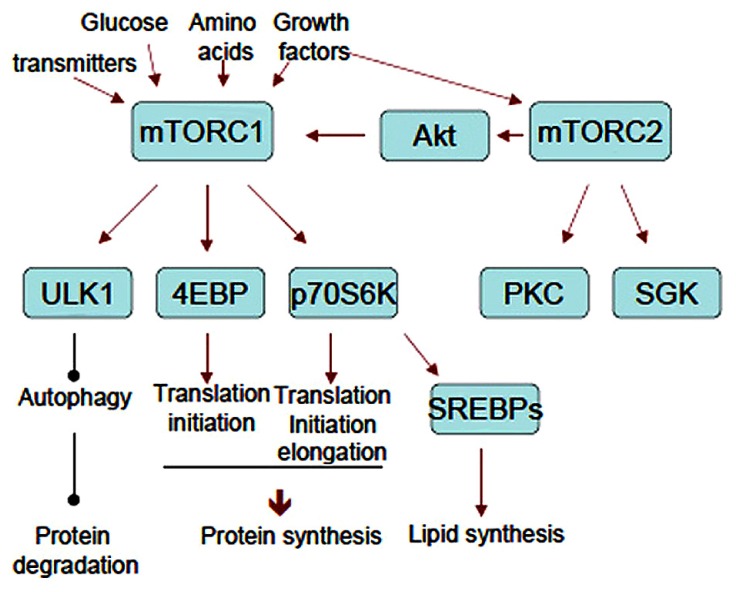
**The flow sheet of upstream and downstream of mTORCs.** Representative substrates and cellular responses mentioned in the text are shown. Note that mTORC2 activates mTORC1 through Akt.

Many substrates lead to diverse cellular responses downstream of mTOR. p70 ribosomal protein S6 kinase (p70S6K) and eukaryotic initiation factor 4E (eIF4E)-binding protein (4EBP) are the best-characterized substrates for mTORC1 that regulate translation. In contrast, ULK1 and ATG13 phosphorylation suppress autophagy. PRAS40, an inhibitory component of mTORC1, is a substrate of mTORC1 itself (Oshiro et al., 2007). All these mTORC1 substrates contain TOR signaling (TOS) motif. TOS motif is recognized by raptor and utilized for substrates recruitment to mTORC1 for optimal phosphorylation (Schalm and Blenis, 2002; Nojima et al., 2003). As mTORC2 substrates, Akt mediates a variety of actions including mTORC1 activation and the inhibition of apoptosis, among others. Protein kinase C (PKC) also mediates a myriad of cellular responses, and serum- and glucocorticoid-induced protein kinase (SGK) affects transcription through FoxO. Recent phosphoproteomic analyses revealed many mTOR substrates that may link to the known cellular responses such as ribosome biogenesis, mitochondrial biogenesis, metabolism, mRNA splicing etc. The flow sheet of mTORCs cascades is summarized in **Figure 2**. The physiological roles of these events in neural functions are less understood. In contrast, translational control mediated by mTORC1 is well studied in the aspect of neural development, plasticity, and diseases. Thus we review this process in detail below.

#### Translational control

**FIGURE 3:**
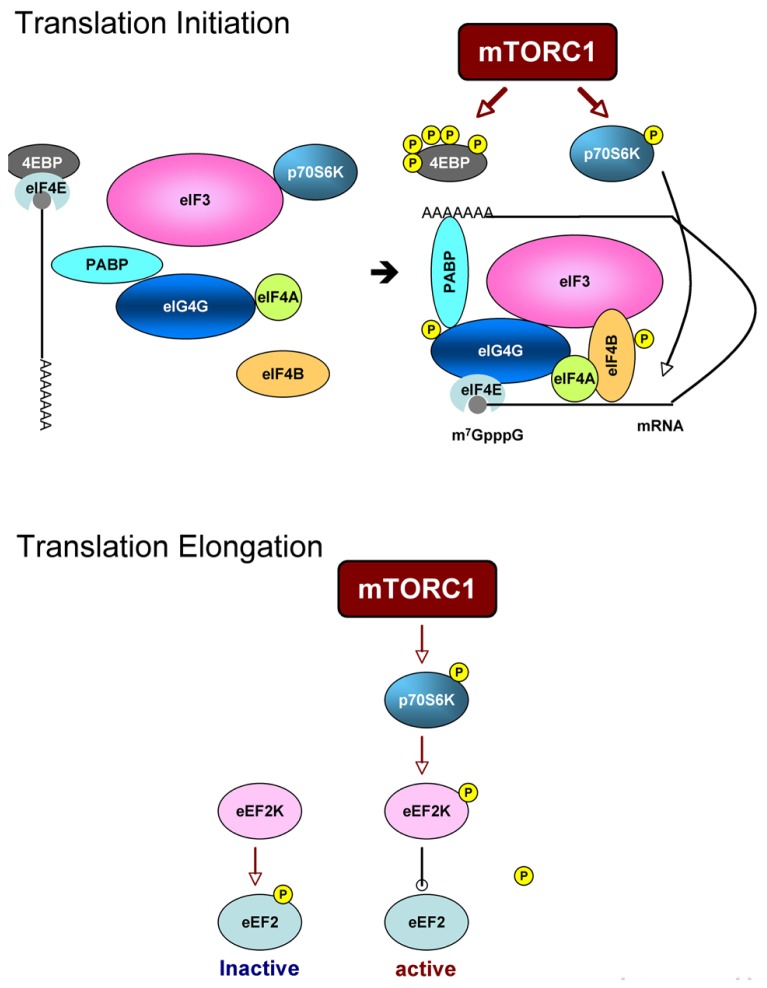
**Scheme of translation processes that are regulated by mTORC1.** Upper panel: translation initiation. mTORC1 directly phosphorylates 4EBP and liberates eIF4E. eIF4E with mRNA then binds to eIF4G to form eIF4F complex. Phosphorylation of eIF4G and eIF4B is mTORC1-dependent. Assembly of eIF3 subunits and eIF4G is also thought to be mTORC1-dependent. Lower panel: translation elongation. p70S6K downstream of mTORC1 phosphorylates eEF2K and suppresses its activity to phosphorylate eEF2. Non-phosphorylated form of eEF2 is an active form thus enhances elongation process.

Upon activation, mTOR phosphorylates p70S6K at Thr389 and 4EBPs [there are three 4EBPs (4EBP1–3), and 4EBP2 is a major isoform in neurons] at Thr37/46 and Ser65 directly. These substrates bind to the scaffold protein raptor by a TOS motif (Schalm and Blenis, 2002; Nojima et al., 2003) and are phosphorylated by the kinase mTOR. Phosphorylation of 4EBPs liberates eIF4E and allows eIF4E to bind to eIF4G and form an eIF4F complex together with eIF4A, an RNA helicase. eIF4E recognizes the 7-methylguanosine 5-triphosphate cap structure of 5′-UTR and poly(A)-binding protein (PABP) binds to the poly(A) tail of mRNAs so that the eIF4F complex makes mRNA circular. Indeed, the circular form of mRNA is thought to stabilize it and facilitate translation. Phosphorylation and activation of p70S6K by mTORC1 induced by insulin facilitates the association of eIF3 (a large molecular complex consisting of 13 subunits) with eIF4G (Holz et al., 2005; Harris et al., 2006). The process is thought to be important for recruiting the 40S ribosome to the mRNA-eIF4F complex. eIF4B is phosphorylated at Ser422 by Akt directly (van Gorp et al., 2009) and by Ser406 and Ser422 in MEK and mTOR/p79S6K-dependent manner (Raught et al., 2004; Shahbazian et al., 2006). Phosphorylated eIF4B enhances eIF4A helicase activity, suggesting that mTORC2 also participates in translational control. eIF4G phosphorylation at Ser1108, Ser1148, and Ser1192 is reportedly rapamycin-sensitive (Raught et al., 2000) (**Figure 3**, Upper panel). In addition, p70S6K phosphorylates eukaryotic elongation factor 2 kinase (eEF2K) and suppresses its activity (Wang et al., 2001), This causes the downregulation of eEF2 phosphorylation thus induces its activation (**Figure 3**, Lower panel).

In neurons, BDNF (Takei et al., 2001, 2004; Liao et al., 2007; Manadas et al., 2009), insulin (Lee et al., 2005; Chenal et al., 2008), and IGF1 (Quevedo et al., 2002; Chenal et al., 2008) have been shown to enhance translation by activating initiation processes through mTORC1 signaling. In addition, BDNF enhances translation elongation processes through mTORC1-dependent downregulation of eEF2 phosphorylation (Inamura et al., 2005). Enhancement of translation by dephosphorylation of eEF2 is also induced by 5-HT (Carroll et al., 2004). A neuron-specific feature of mTORC1-dependent translational control is its spatial property. For example, the mTORC1 system is localized in dendrites as well as in cell bodies (Takei et al., 2004). BDNF (Takei et al., 2004) and transmitters (Casadio et al., 1999; Carroll et al., 2004; Gong et al., 2006) have been shown to induce “local” activation of mTORC1 and translation (see review in this issue).

In summary, mTORC1 (and possibly mTORC2) modulates translational processes by the following: mTOR 1) phosphorylates 4EBP and induces cap-dependent translation; 2) phosphorylates p70S6K and allows eIF3 to bind eIF4G; 3) induces eIF4B phosphorylation through p70S6K and Akt; and 4) activates eEF2 through p-70S6K/eEF2K. Therefore, mTOR regulates translation both at initiation and elongation steps.

A recent report suggested that mTORC1 specifically controls the translation of a certain subset of mRNAs (Thoreen et al., 2012). eIF4E preferentially binds to mRNAs that harbor 5′-terminal oligopyrimidine tract (5′-TOP) or related sequences. These mRNAs encode proteins of translation machinery such as ribosomal proteins and translation factors. It has been postulated that p70S6K/S6 regulates translation of 5′-TOP mRNAs. However, although these events occur in parallel, genetic and biochemical analysis have refuted a direct relation between them (Shima et al., 1998; Ruvinsky and Meyuhas, 2006). This new insight can explain the direct interaction of mTORC1 and 5′-TOP mRNA translation. Indeed, acute signal(s) that activates mTORC1 enhances the production of proteins necessary for translation machinery and may prepare the succeeding and continuous activation of translation that leads to cellular growth. The authors concluded that 4EBP and eIF4G were the master regulators of mTORC1-dependent translation. However, further research must be conducted in postmitotic neurons, because the elongation process was the rate limiting step for translation in neurons (Takei et al., 2009).

Many reports have suggested that mTORC1-dependent translational control is crucial in the development and plasticity of neurons. However, in the majority of the studies, conclusions are based on pharmacological approaches using inhibitors. Rapamycin, the most commonly used tool for analyzing mTORC1 signals, does not effectively inhibit all mTORC1-dependent translation (Kang et al., 2013). An ATP-competitive mTOR inhibitor such as Torin1 both inhibits rapamycin-resistant mTORC1 reactions and mTORC2 activity. Chronic rapamycin also inhibits mTORC2. The effectiveness is somewhat cell-type specific, and the efficacy of rapamycin on mTORC2 in neurons has not yet been verified. In any case, we suggest caution when making conclusions using simple pharmacological approaches. Although it is technically challenging, monitoring the actual translation processes together with analysis of translation machinery as well as mTORC1 signaling is desired in combined with certain biological/physiological responses.

#### Lipogenesis

In addition to protein synthesis, mTORC1 also enhances lipid biosynthesis (see review by Laplante and Sabatini, 2009). mTORC1 has been reported to activate SREBP (sterol regulatory element binding protein) 1 and 2, transcription factors responsible for the expression of many lipid metabolic enzymes (Düvel et al., 2010). SREBPs are cleaved and the processed forms are translocated to the nuclei and promote gene transcription. Transcriptome and metabolome analysis have shown that SREBP-dependent lipid biosynthesis is rapamycin-sensitive (Düvel et al., 2010). Rapamycin inhibited lipid biosynthesis and the expression of lipid synthetic enzymes including acetyl-CoA carboxylase (Brown et al., 2007), fatty acid synthase (Peng et al., 2002), and HMG-CoA reductase (Yamauchi et al., 2011). Fatty acid oxidation (Brown et al., 2007) and cholesterol synthesis (Yamauchi et al., 2011) have also been shown to be rapamycin-sensitive. A recent report showed that mTORC1-mediated activation of SREBP1 and 2 was mediated by p70S6K (Düvel et al., 2010). Deletion of p70S6K induces the small body and small cell phenotype but does not affect translation; thus, the phenotype may be the result of inhibition of lipid synthesis, rather than protein synthesis. Because lipids are necessary to form plasma and organelle membrane component, as well as energy storage source and intracellular signaling, there is no doubt that lipid synthesis is essential to cell growth. Indeed, SREBP1 knockdown, as well as rapamycin treatment, can reduce cell size (Porstmann et al., 2008). Thus, both protein and lipid synthesis controlled by mTORC1 must be necessary for cell size regulation.

In the CNS, BDNF, the most potent activator of mTORC1 in neurons, has been shown to enhance cholesterol synthesis (Suzuki et al., 2007), as well as protein synthesis. Thus, mTORC1 must contribute to BDNF-mediated neuronal cell growth and dendritic arborization (e.g., McAllister et al., 1995, see review by Lewin and Barde, 1996). Cancer cells that exhibit uncontrolled growth factor signaling often show activation of SREBPs and lipogenesis. Enough (or excess) lipids required for membrane synthesis are a critical process of cancer progression including migration and invasion along with membrane expansion. These cellular responses give us insight into the characteristics of neuritis extension and spine formation during development and synaptic plasticity. Thus, BDNF-mediated neuronal growth may be dependent, at least in part, on de novo lipid synthesis.

## BRAIN DEVELOPMENT AND mTOR

### ROLES OF mTOR IN NORMAL BRAIN DEVELOPMENT

mTOR plays a pivotal role in growth, proliferation, and migration of every cell; thus, it is thought to be essential for the development of an organism. Indeed, a knockout (KO) mouse study showed that mTOR was indispensable for normal development and viability (Murakami et al., 2004). The first genetic evidence that mTOR plays an important role in brain development came from an ethyl-nitroso-urea-induced mouse mutation screening (Hentges et al., 2001). This mutant, named flat-top, was a loss of function mutant of mTOR by missplicing; it showed a defect in telencephalon formation and died in mid-gestation. The milder phenotype compared to full KO may result from the incomplete loss of mTOR function. Indeed, the mutant still had about 10% p70S6K activity compared to the wild-type mouse.

Complete ablation of mTORC components in mice resulted in embryonic lethality (Guertin et al., 2006; Shiota et al., 2006). In addition to mice lacking mTOR itself, raptor KO mice died very early in development, while mLST8 or rictor-null mice died at embryonic day 10.5. Therefore, a conditional KO (CKO) strategy was applied for analyzing the function of mTORCs in brain. Elimination of mTORC1 function in brain was achieved by crossing raptor-floxed mice and nestin-Cre mice (Cloëtta et al., 2013). Nestin is a marker of neural progenitors. Although the raptor-CKO mice died soon after birth, they showed microcephaly via reductions in cell size and cell number. In addition, gliogenesis was affected in parallel with the downregulation of STAT3 phosphorylation at Ser727. This is plausible because STAT3 is pivotal for gliogenesis (Bonni et al., 1997) and is phosphorylated by mTORC1 (Yokogami et al., 2000).

The conditional deletion of rictor in several tissues shows a relatively mild phenotype compared with that of raptor. Mice with brain-specific deletion of rictor by crossing nestin-Cre mice have been shown to survive but with smaller brain size, like raptor-KO mice (Thomanetz et al., 2013). Brain weight of this rictor-KO mouse was about 60% (nestin-Cre) of the wild-type mouse brain. Total dendritic lengths and soma sizes of pyramidal neurons of hippocampus and Purkinje neurons of cerebellum were reduced. Purkinje cell-specific deletion of rictor via an L7/Pcp-2-cre mouse showed impairments in synaptic function and morphology of these neurons, which are correlated with the ataxia-like phenotype of this mouse (Thomanetz et al., 2013). Another report employed Emx1 promoter to drive Cre (Carson et al., 2013). Emx1 expresses only in dorsal neural progenitor cells that generate excitatory neurons and astrocytes in the dorsal cortex. The Emx1-Cre:rictor CKO mice also showed a small brain but to a lesser extent (about 80% of the wild-type mice), because the rictor-deficient cell population was more limited than that in the nesti-Cre:rictor mice. Neurons in cortical layers II–IV of the Emx1-Cre:rictor conditional mice were smaller than that of wild-type mice. These results implicate mTORC2 in the progression of neuronal size and morphology during brain development. To put it simply, because mTORC2 activates Akt, it will cause mTORC1 activation. If mTORC2 is inhibited, mTORC1 activity may be downregulated. However, mTORC1 activity looked normal in this rictor CKO mouse. In addition, in other cell types in other organs, for example muscle cells, mTORC2 inactivation has little effect on cell size compared to the mTORC1 inactivation (Bentzinger et al., 2008). The precise mechanism of how mTORC2 regulates cell size is still unresolved.

### ROLES OF mTOR IN DENDRITE FORMATION

Detailed morphological analysis of mTOR on neurite formation comes from a culture study. Specifically, transfection of constitutively active or dominant negative forms of PI3K, Akt, and Ras revealed that PI3K-Akt increased the size of the soma and dendrites (Jaworski et al., 2005). Ras combined with PI3K-Akt enhanced the complexity of dendrites of hippocampal neurons (Kumar et al., 2005). Similarly, knockdown of phosphatase and tensin homolog (PTEN), the phosphatase for Akt, by siRNA induced arborization of hippocampal dendrites (Jaworski et al., 2005). The dendritic growth induced by these manipulations was antagonized by chronic rapamycin treatment, siRNA-mediated knockdown of mTOR and p70S6K, and overexpression of phosphorylation-defective mutant 4EBP (Jaworski et al., 2005). These results indicate that mTOR, especially mTORC1, play pivotal roles in dendritic growth/maturation. Mice with neuron-specific deletion of PTEN showed macrocephaly and hypertrophy of neurons with enhanced mTORC1 signaling (Kwon et al., 2006). Enhanced mTORC1 signaling and dendritic arborization by extracellular stimuli like BDNF (Jaworski et al., 2005) and Reelin (Jossin and Goffinet, 2007) are both inhibited by rapamycin. In addition to mTORC1, mTORC2 is implicated in the dendritic growth of hippocampal neurons. The dendritic arbor was disturbed not only by raptor but also by rictor small hairpin (sh) RNA, as well as by mTOR inhibitor Ku-006379A, which inhibits both mTORC1 and 2.

### ROLES OF mTOR IN AXON ELONGATION

Axon guidance during development is regulated by balanced chemotactic cues by attractive and repulsive molecules. Semaphorin-3 and netrin-1 have been shown to induce growth cone collapse and repulsive turning in *Xenopus* retinal neurons. The response was abrogated by rapamycin, as well as by protein synthesis inhibitors, cycloheximide and anisomycin. Phosphorylation of 4EBP was observed in growth cones in response to semaphorin-3 and netrin-1 (Campbell and Holt, 2001). Slit2 has been shown to act on growth cone collapse and 4EBP phosphorylation similarly, but at later stage, in a rapamycin-sensitive manner (Piper et al., 2006). TSC2 haploinsufficiency in mice (TSC-/+) caused aberrant retinogenicular projection, suggesting the disruption of axon guidance. Since axon guidance of this tract is known to depend on ephrin-Eph signaling, the effect of ephrinA on mTOR pathway was investigated. In contrast to the case with semaphorin-3, the ephrinA-EphA signal that induced growth cone collapse suppressed the MAPK-TSC2-mTORC1 cascade and inhibited novel protein synthesis. TSC deficiency and constitutively active Rheb expression have been reported to counteract the actions of ephrinA-EphA (Nie et al., 2010). These results indicate that mTORC1-mediated translational control of growth cones plays pivotal roles in axon guidance during development.

These studies reveal the universal and specific roles of mTORCs in neurons. mTORC1 activation induces protein and lipid synthesis so that it increases cellular mass with expansion of plasma membrane. Local protein (and possibly lipid) synthesis mediated by mTORC1 may participate in the extension of an axon and dendrites of a neuron. mTORC2 may be the a putative facilitator of growth cone motility, including neurite pathfinding and elongation because it is known to affect actin dynamics. Although the precise mechanisms are still not fully understood, regulated and coordinated activities of mTORC1 and 2 must be necessary for normal development of neurons and a brain.

## BRAIN PATHOLOGY CAUSED BY mTORC1 SIGNALING ABNORMALITY

### TUBEROUS SCLEROSIS (TSC)

mTOR has been implicated as a cause of various diseases. Among them, most famous and directly related to mTOR disease is TSC. As mentioned previously, this disease is caused by the genetic mutation (including nonsense, missense, insertion, and deletion) of *TSC1* or *TSC2*, the genes encoding TSC1 (hamartin) or TSC2 (tuberin), respectively. TSC was first described in late 19th century and the mutations have been identified in *TSC1* (9q34) and *TSC2* (16p13.3) in the 1990s. In 2002, several groups reported that TSC1/2 complex is a suppressor of mTOR. TSC1/2 complex is a GAP for Rheb so it inhibits mTORC1.

Tuberous sclerosis complex is an autosomal dominant, multisystem disorder that affects brain, lung, heart, skin, and kidney. The neurological symptoms are intractable epilepsy, autism, and mental retardation. Pathological features are cortical tubers, subependymal nodules (SENs), and subependymal giant cell tumors (SGCTs) and subependymal giant cell astrocytomas (SEGAs). The characteristic features are abnormal cell proliferation and growth (see review Crino et al., 2006; Crino, 2013). Because TSC is caused by a loss-of-function of TSC1 or TSC2, mTORC1 must be constitutively active in these cells. Indeed, highly phosphorylated p70S6K (Thr389), S6 (Ser235/236), and 4EBP (Thr37/46) have been observed immunohistochemically in pathologically abnormal, enlarged cells that may be of progenitor cell origin (Tsai et al., 2012; Prabowo et al., 2013). Although the direct interaction of TSC1/2 and mTORC2 is still unclear, phosphorylation of mTORC2 substrates PKCa at Ser657, Akt at Ser473, and SGK1 at Ser422 have also been observed in the fetal TSC brain (Tsai et al., 2012). The relationship between the activation of mTORC1 and cell (over)growth observed in human disease corroborates the experimental findings. In fact, the rapamycin analogue (called “Rapalog”) everolimus (RAD-001) is clinically approved for the medication of TSC (http://www.fda.gov/Drugs/InformationOnDrugs/ApprovedDrugs/ucm317490.html).

### ANIMAL MODELS OF TSC

There have been many reports about the KO of *TSC1* or *TSC2* genes. Homozygotes of these gene deficiency are lethal, and heterozygotes do not have tubers, although the mice show some abnormalities. Several CKO mice carrying biallelic deletion in certain cells have been generated, and they mimic some symptoms and pathology of human TSC. This may be because biallelic mutation is necessary to induce severe morphological alteration such as tubers or giant astrocytomas. In fact, so-called “two-hit” mechanisms of germline mutation and somatic mutation have been proposed for human tubers of TSC patients (Crino et al., 2010), although another report argued that the second hit was rare (Qin et al., 2010). The two-hit mechanism has been evaluated with homozygous deletion of *TSC2* only in radial glia, and heterozygous deletion in all other cells by crossing Tsc2+/-, Tsc2flox, and GFAP-Cre mice (Way et al., 2009). The mice showed cytomegaly, defects in lamination, astrogliosis, and hypomyelination. mTORC1 activation was confirmed by S6 phosphorylation. Another approach of double hit of *TSC1* gene by in utero electroporation has been reported. The mice were made by crossing TSC1+/- mice and TSC1flox mice. TSC1 in the cells of certain brain regions were biallelically deleted via in utero electroporation of Cre plasmid. The offspring showed tuber-like lesions and cortical hyperexcitability (Feliciano et al., 2011). Other conditional homozygotic deletion of either TSC1 or 2 represented some of the features of TSC (Magri et al., 2011). All the animal models indicate that biallelic deletion (or dysfunction) is necessary to induce TSC pathology. We have learned a lot about mTOR biology from TSC including the signaling mechanism of mTORCs; however, there are still many unsolved questions like the above-mentioned two-hit hypothesis. Further comprehensive genetic analysis (not limited in *TSC* genes) in the TSC brain may address the pathological obscurity of genotype-phenotype interaction.

### DISEASES PATHOLOGICALLY RELATED TO TSC

There are two diseases with neurological symptoms and brain pathology similar to TSC – hemimegalencephaly and focal cortical dysplasia. Genetic mutations of these diseases have not been identified until now. Recently, somatic mutations of Akt, PI3K, and mTOR were reported in the hemimegalencephaly brain (Lee et al., 2012; Poduri et al., 2012). Two of the cases are a trisomy of 1q that contains Akt3, and one is an activating mutation of Akt3 (G49A; Poduri et al., 2012). This mutation encompasses the amino acid substitution E17K in a coding region, which converts Akt to a constitutively active form. Another group found same somatic mutation of Akt3 in one case, and constitutively active PI3K mutations (G1633A, E545K) in four cases. In addition, an mTOR somatic mutation at C4448T that causes C1483Y substitution was observed in hemimegalencephaly brain (Lee et al., 2012). In contrast to some mTOR active mutations developed in laboratories in mammalian cell (Urano et al., 2007; Ohne et al., 2008; Hardt et al., 2011), whether C1483Y mutation makes mTOR active or not is unknown. This is the first report of mTOR mutation in neurological disease so that the validation of activity is awaited. Dysmorphic cells in the brain of focal cortical dysplasia type IIB patient showed hyper-phosphorylation of S6 (Baybis et al., 2004; Miyata et al., 2004). Although genetic analysis forthcoming, it is likely that there may be somatic mutations of unknown genes in mTORC1 signaling pathways that are responsible for cellular abnormality in focal cortical dysplasia.

## CONCLUSION AND FUTURE PERSPECTIVE

In this review, we summarize the updated molecular interaction of mTOR signaling. We focus on the growth and size regulation of neurons during development as a biological output of mTOR signaling. Diseases that are picked are limited to this aspect. TSC is caused by the mutation of TSC1 or 2 that are regulators of mTORC1. TSC and other pathologically related disease, hemimegalencephaly and forcal cortical dysplasia are characteristics of enlarged and dysmorphic neurons and glia. These are diseases of dysregulated cell growth.

Although mTOR governs various processes, we describes much on the translational control and a bit about lipid biogenesis, because these two processes seem to be related with cell growth regulation directly. In addition, mTOR-mediated translational control is most (or almost only) studied in the field of neuroscience as a mechanism behind the neural plasticity, learning and memory.

Pathology of TSC has given us a hit of link between mTORC1 activity and size control in the brain. Overactivation of mTORC1 (and potentially mTORC2) induces enlargement and dysmorphism of neurons and glia (or their progenitors). On the other hand, it has been well known that fetal and neonatal malnutrition causes reduced brain size (Morgane et al., 1993). Insufficient intake of proteins and carbohydrates causes reduced amino acid and glucose. Because these nutrients are essential factors to induce mTORC1 function, it is likely that mTORC1 inhibition is related to dysgenesis of a brain. Beside these unusual situations, size differences are found in neurons. One characteristic feature of neurons is its high heterogeneity in function and morphology. Soma sizes vary from about 10 (cerebellar granule neurons) to 100 (Betz cell) micrometers in diameter. The cell size is thought to be determined genetically (and/or epigenetically). The concept includes timing and levels of expression of nutrients transporters and growth factor receptors that may affect mTORC1 activity. Although mTOR signaling components are ubiquitously expressed, rheb, for example, is an activity-inducible molecule in neurons. It suggests that neural activity may enhance mTORC1 through the upregulation of rheb level.

**FIGURE 4:**
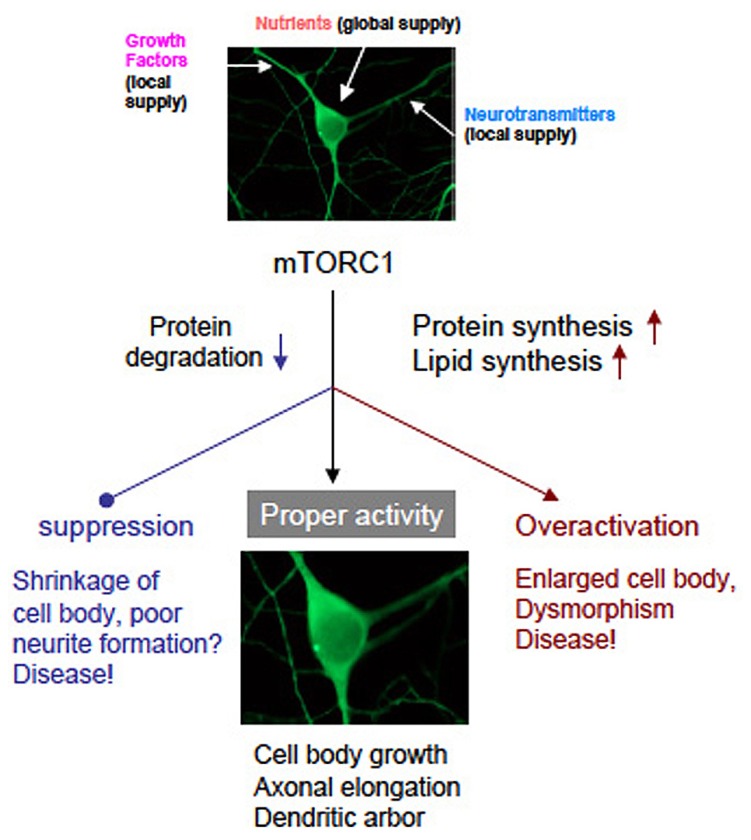
**A graphic of hypothetical neuronal development governed by mTORC1.** Neurons receive nutrients globally and growth factors/transmitters locally. Both inputs coordinately activate mTORC1 that leads normal neuronal development. Suppression or overactivation of mTORC1 result dysregulation of neuronal morphology and function. (note that photographs of a neuron was image processed).

mTOR is undoubtedly a master regulator of cell growth from yeast to human. However, how mTOR controls cell size is still not clear enough. Enhanced protein and lipid synthesis, and inhibition of protein degradation controlled by mTORC1 surely increase cellular mass that includes cell volume, axon elongation, and dendrite arborization. Conceptual scheme is shown in **Figure 4**. It must be clarify whether novel protein and/or lipid synthesis are necessary and sufficient for cell growth in the brain. Further studies will reveal the correspondence to mTOR downstream signaling pathway to phenotype (cell growth). It will shed light on the biology of cell size and may also contribute to the drug discovery for the TSC and related diseases more selective than rapamycin and rapalogs.

## Conflict of Interest Statement

The authors declare that the research was conducted in the absence of any commercial or financial relationships that could be construed as a potential conflict of interest.
